# Supplementary figures and images for: Growth and development of succulent mixtures for extensive green roofs in a Mediterranean climate
Source: PLoS One. 2022 Jun 3;17(6):e0269446. doi: 10.1371/journal.pone.0269446 (PMC9165813; doi:10.1371/journal.pone.0269446)

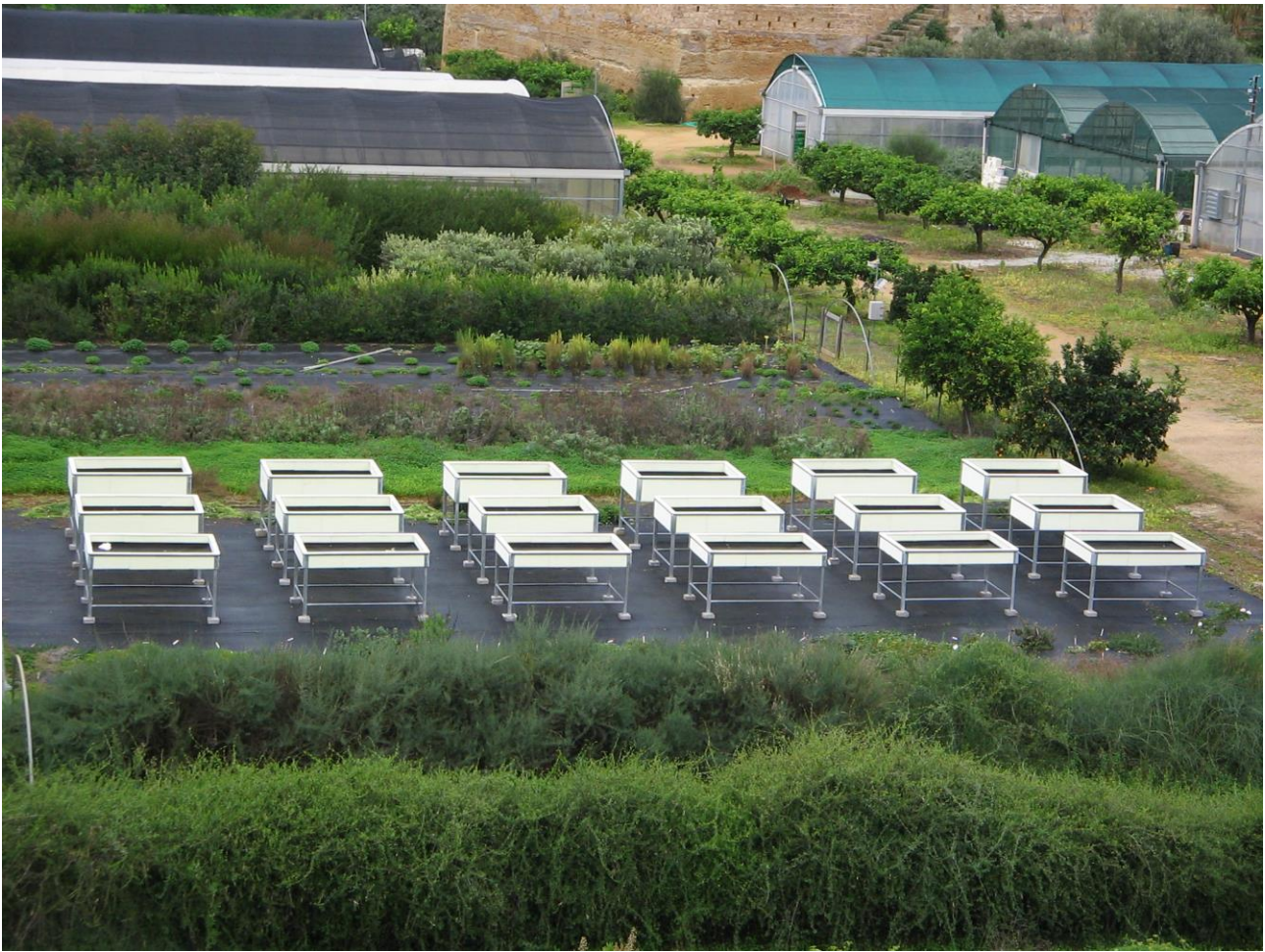

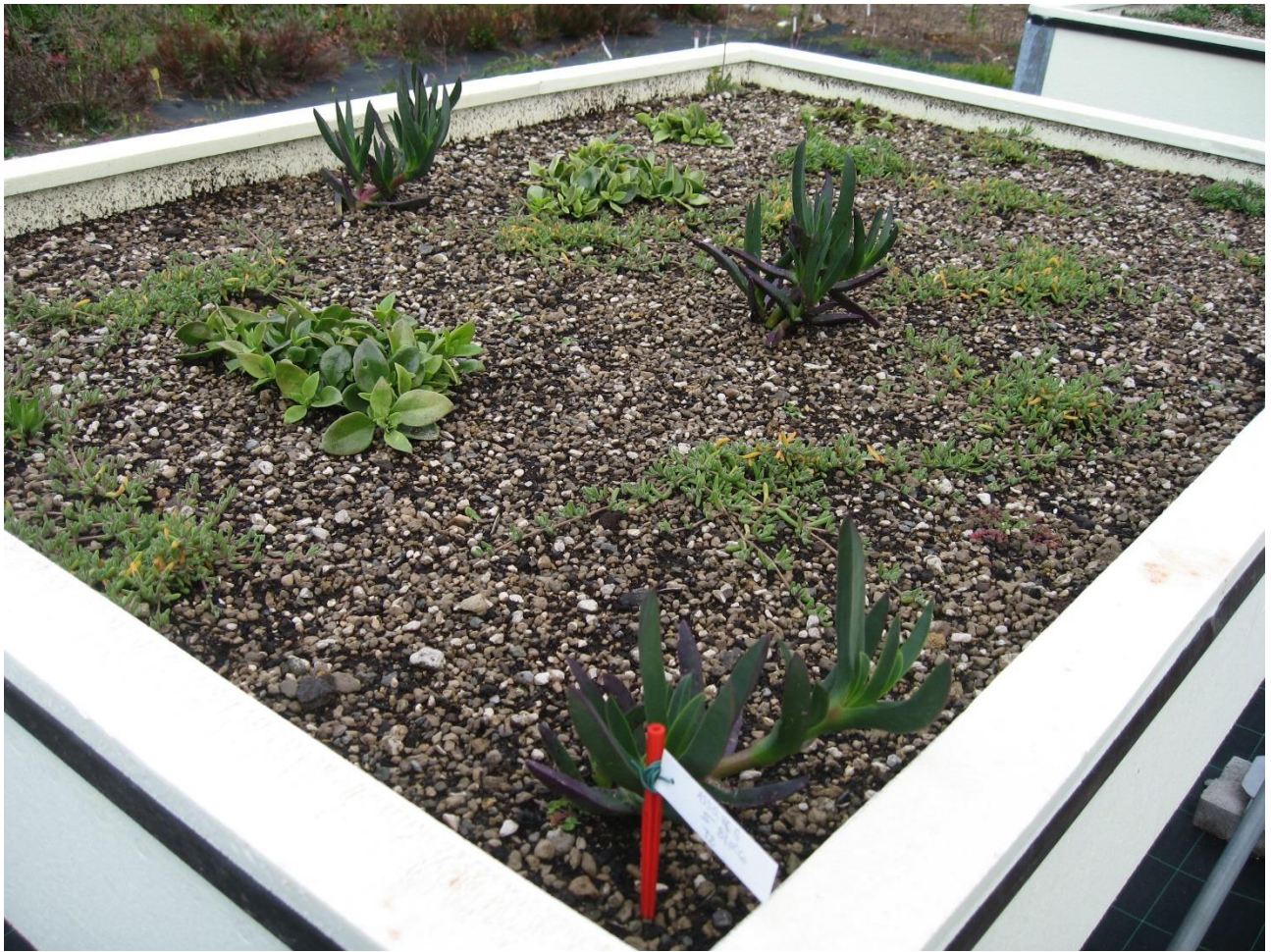

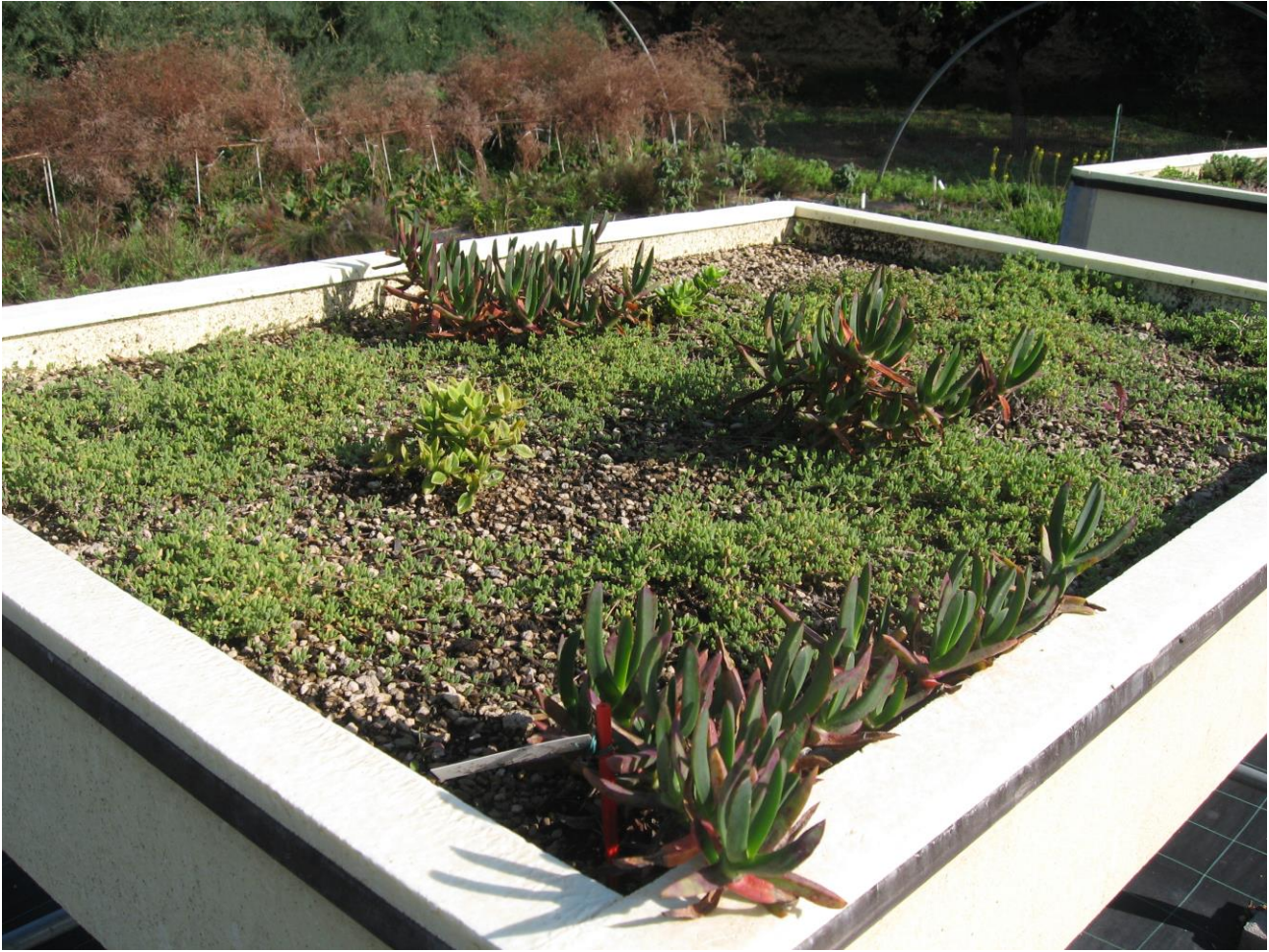

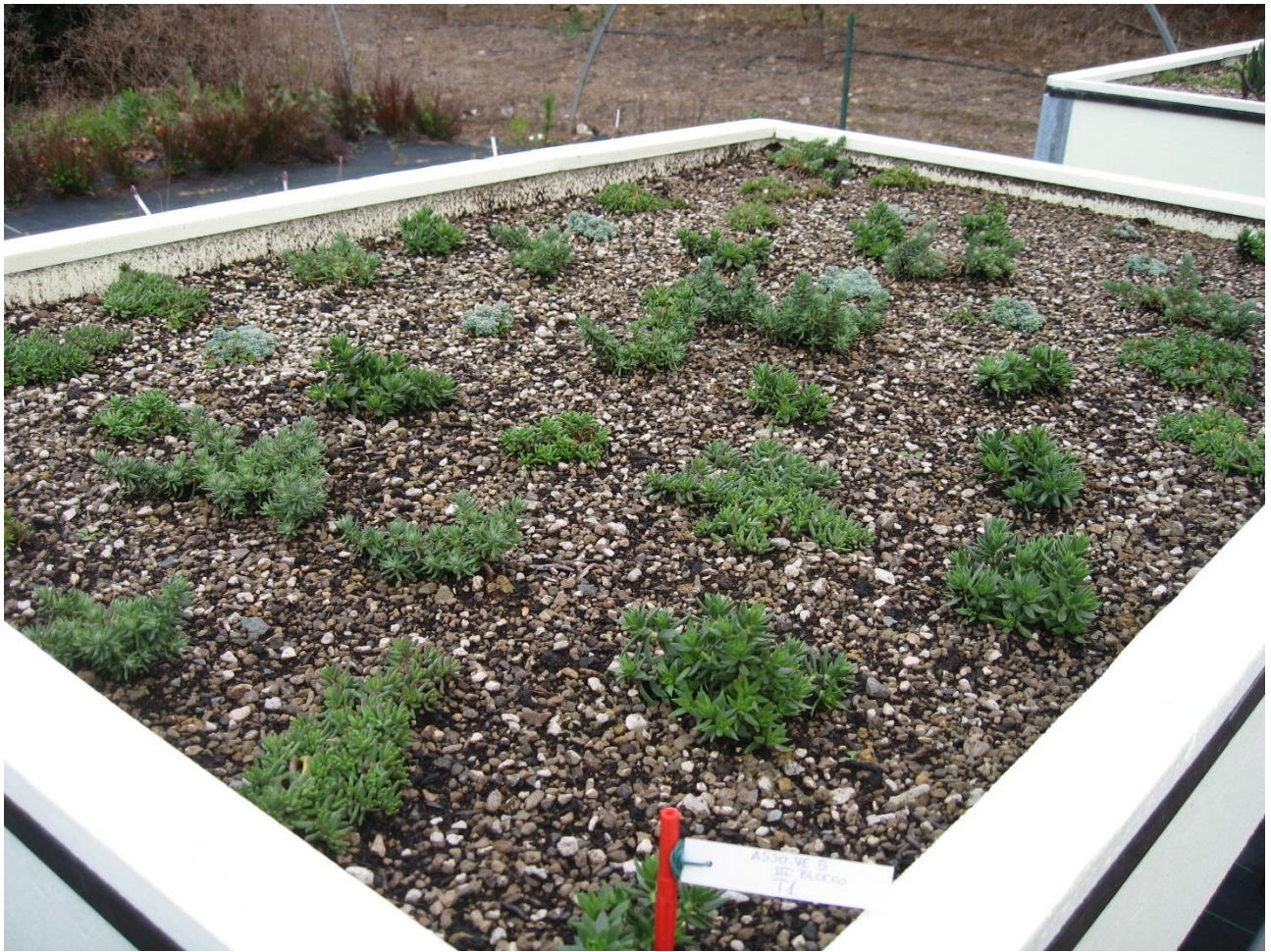

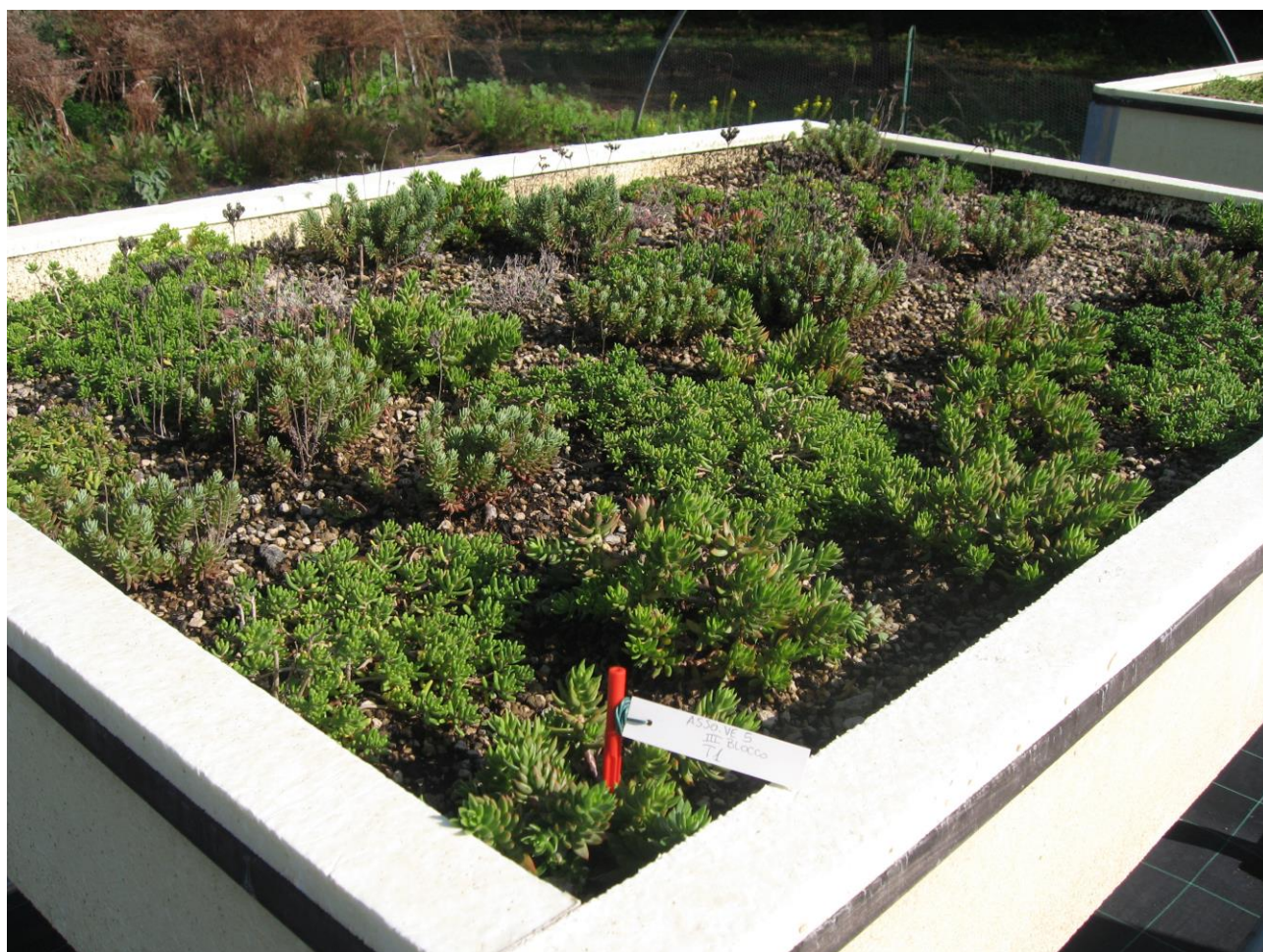

Supplement: S1 Fig — (PDF) [file pone.0269446.s001.pdf]
